# The effect of education participation on youth custody: Causal evidence from England

**DOI:** 10.23889/ijpds.v8i2.2214

**Published:** 2023-09-14

**Authors:** Matt Dickson

**Affiliations:** 1 University of Bath, Bath, United Kingdom

## Objectives

This paper estimates the effect of continuing in education post-16 on the probability of experiencing youth custody at ages 17 and 18, addressing the issue of non-random selection into continued participation to derive a causal estimate.

## Method

We exploit the natural experiment created by the ‘raising of the participation age’ (RPA) in England. Unlike previous cohorts who could leave education aged 16, young people starting the final year of compulsory schooling in September 2012 were required to continue in education or training until the end of the school year in which they turned 17, and those starting the final year in September 2013 were required to continue until age 18. Using linked National Pupil Database and National Client Caseload Information System data we utilise the variation in participation between cohorts that the RPA induced to estimate the causal effect of continued participation on custody outcomes at ages 17 and 18.

## Results

The effect of the law change was to increase the proportion of young people participating in education at age 17 by 1.7pp (1.2pp) for boys (girls), from a base of 82.1\% (85.0\%) prior to the reform. Despite this increase in participation, there was no effect on the probability of custody when aged 17 or 18. This suggests that the 0.64pp (0.04pp) reduction in probability of custody associated with continued participation for boys (girls) estimated without addressing the selection issue, is actually capturing the effect on custody probability of the unobservable characteristics of those who choose to continue in education beyond 16. Results are robust to different estimation methods and different treatment specifications.

## Conclusion

 The negative relationship between education and crime is well documented but the decision to remain in education beyond the compulsory age is not random. Evidence here suggests that the cross-sectional reduction in probability of custody associated with continued education is driven by the unobservable characteristics of those who voluntarily continue their education rather than reflecting a causal effect of education.

